# Supplementary material for: Landscape Enhancements in Apple Orchards: Higher Bumble Bee Queen Species Richness, but No Effect on Apple Quality
Source: Insects. 2021 May 8;12(5):421. doi: 10.3390/insects12050421 (PMC8151366; doi:10.3390/insects12050421)
Supplement: Supplementary file 1 [file insects-12-00421-s001.zip › insects-1185100-supplementary.pdf]

Table S1. List of pesticides used at the 12 orchards sampled in 2017 in Southern Québec, Canada. Information on the active ingredient(s), the usage and the bee toxicity were taken from SAgE pesticides web site [1]. When bee toxicity was unknown, the product was not included in the formula estimating the intensity of pesticide use on a given orchard (see Methods). The last column indicates if the pesticide was only used in orchard without landscape enhancement (Without), only in orchards with landscape enhancement (With) or in both (Both).

**Commercial Name**	**Active Ingredient**	**Usage**	**Bee Toxicity**	**Found in orchards**
Acramite[2]	Bifenazate	Insecticide	Medium	Without
Agral[2]	Nonylphenoxypolyethoxyethanol	Adjuvant	Not Known	Without
Agri-Mek[2]	Abamectine	Insecticide	High	Without
Altacor*	Chlorantraniliprole	Insecticide	Low	Both
	Ammonium Sulfate[2]	Fertilizer	Low	Without
Apogee[2]	Prohexadione-Calcium	Growth Regulator	Not Known	Without
Assail[2]	Acetamiprid	Insecticide	Medium	With
Beleaf[1]	Flonicamid	Insecticide	Low	Both
	Potassium Bicarbonate *	Fongicide	Low	Both
	Boron*	Fertilizer	Low	Both
	Calcium Polysulfide*	Fongicide And Insecticide	Low	Both
Caltrac*	Calcium	Fertilizer	Low	Both
Calypso*	Thiacloprid	Insecticide	Low	Both
Captan*	Captan	Fongicide	Low	Both
Clutch[2]	Clothianidin	Insecticide	High	Without
	Copper*	Fongicide	Low	Both
Decis[2]	Deltamethrin	Insecticide	High	Without
Dipel[1]	Bacillus thuringiensis subsp. kurstaki Strain ABTS-351	Insecticide	Low	With
Entrust*	Spinosad	Insecticide	High	Both
Envidor[2]	Spirodiclofen	Acaricide	Low	Without

Exirel[1]	Cyantraniliprole	Insecticide	High	With
Flint[1]	Trifloxystrobine	Fongicide	Low	With
Fontelis*	Penthiopyrad	Fongicide	Low	Both
Fruitone*	1-Naphthaleneacetic Acid	Growth Regulator	Low	Both
Gf-120 Fruit Fly Bait*	Spinosad	Insecticide	High	Both
Glyphos[1]	Glyphosate	Herbicide	Low	With
Imidan*	Phosmet	Insecticide	High	Both
Inspire Super[1]	Cyprodinil / Difénoconazole	Fongicide	Low	With
Isomate[1]	Pheromones	Attractive	Low	With
Kumulus*	Sulfur	Fongicide	Low	Both
Li-700*	Free Fatty Acids And IPA	Adjuvant	Low	Both
Mako[2]	Cypermethrin	Insecticide	High	Without
Manganese[1]	Manganese		Low	With
Manzate*	Mancozeb	Fongicide	Low	Both
Movento*	Spirotetramat	Insecticide	Low	Both
Nealta[2]	Cyflumetofen	Acaricide	Low	Without
Nova*	Myclobutanil	Fongicide	Low	Both
Polyram*	Metiram	Fongicide	Low	Both
	Potassium Bicarbonate *	Fongicide	Low	Both
Retain*	Thifensulfuron-Methyl / Tribenuron-Methyl	Herbicide	Medium	Both
Ripcord[1]	Cyperméthrin	Insecticide	High	With
Roundup Transorb[1]	Glyphosate	Herbicide	Low	With
Scala*	Pyrimethanil	Fongicide	Low	Both
Sel D'epson*	Magnesium		Low	Both
Sevin[1]	Carbaryl	Insecticide	High	With
Steptomycine*	Streptomycin	Bactericide	Low	Both
Superior 70 Oil*	Mineral Oil	Insecticide	Low	Both
	Urea*	Fertilizer	Low	Both

Virosoft[2]	Codling Moth Granolosis Virus	Insecticide	Not Known	Without
Xiameter*	Siloxylated Polyether	Adjuvant	Not Known	Both
	Zinc[2]	Fertilizer	Low	Without

Table S2. Formulation of Bayesian hierarchical community occupancy model implemented in JAGS for the bumblebee queen data collected between 2017 and 2019 in southern Québec, Canada.

```
model{
##prior distribution of average occupancy over all species in the community
psi.mean ~ dunif(0, 1)
beta0 <- log(psi.mean) - log(1 – psi.mean)

##prior distribution of average detection over all species in the community
p.mean ~ dunif(0, 1)
alpha0 <- log(p.mean) - log(1 - p.mean)

##prior distribution of beta parameters for covariates on occupancy
mua1 ~ dnorm(0, 0.001) #hyperparameter defining mean of random slope of season
mua2 ~ dnorm(0, 0.001) #hyperparameter defining mean of random slope of management
mua3 ~ dnorm(0, 0.001) #hyperparameter defining mean of random slope of intensity

##prior distribution of beta parameters for covariates on detection
nub1 ~ dnorm(0, 0.001) #hyperparameter defining mean of random slope of airtemp
nub2 ~ dnorm(0, 0.001) #hyperparameter defining mean of random slope of time of day

##prior distribution of SD of average occupancy
sigma.u ~ dunif(0, 50)

##prior distribution of SD of average detection
sigma.v ~ dunif(0, 50)

##precision (1/variance) of parameters
tau.u <- pow(sigma.u, -2)
tau.v <- pow(sigma.v, -2)

##variance of beta parameters on occupancy
sigma.a1 ~ dunif(0, 50) #hyperparameter defining variance of random slope of season
tau.a1 <- pow(sigma.a1, -2)
sigma.a2 ~ dunif(0, 50) #hyperparameter defining variance of random slope of management
tau.a2 <- pow(sigma.a2, -2)
sigma.a3 ~ dunif(0, 50) #hyperparameter defining variance of random slope of intensity
tau.a3 <- pow(sigma.a3, -2)

##variance of beta parameters on detection
```

```
sigma.b1 ~ dunif(0, 50) #hyperparameter defining variance of random slope of airtemp
sigma.b2 ~ dunif(0, 50) #hyperparameter defining variance of random slope of time of day
tau.b1 <- pow(sigma.b1, -2)
tau.b2 <- pow(sigma.b2, -2)

##random effect of site on psi
sigma.psi.site ~ dunif(0, 150)
tau.psi.site <- pow(sigma.psi.site, -2)
for (b in 1:nsiteID){
  alpha.site[b] ~ dnorm(0, tau.psi.site)
  }
for (i in 1:nspecies) {

##Create priors for species i from the community level prior distributions
   ##parameters on occupancy
   phi0[i] ~ dnorm(beta0, tau.u) #intercept on occupancy
   a1[i] ~ dnorm(mua1, tau.a1) #beta for season
   a2[i] ~ dnorm(mua2, tau.a2) #beta for management
   a3[i] ~ dnorm(mua3, tau.a3) #beta for intensity (low = 0 vs high = 1)

   ##parameters on detectability
   eta0[i] ~ dnorm(alpha0, tau.v) #intercept on detection
   b1[i] ~ dnorm(nub1, tau.b1)    #beta for airtemp
   b2[i] ~ dnorm(nub2, tau.b2)    #beta for time of day

##Loop to estimate the Z matrix (true occurrence for species i at site j
  for (j in 1:nsite) {
     logit(psi[j, i]) <- phi0[i] + a1[i]*Season[j] + a2[i]*Management[j] + a3[i]*Intensity[j] + alpha.site[SiteNum[i]]
        Z[j, i] ~ dbern(psi[j, i]) #prior distribution for latent variable of species i in site j
     ##Loop to estimate detection for species i at site j during sampling period k.
     for (k in 1:nvisit) {
     logit(p[j, k, i]) <-  eta0[i] + b1[i]*Airtemp[j, k] + b2[i]*Time[j, k]
     ##if species not present (Z = 0), then p = 0 for species (species cannot be detected)
     mu.p[j,k,i] <- p[j, k, i]*Z[j, i]
     y[j,k,i] ~ dbern(mu.p[j, k, i])
}
}
}

##species richness at each site
for(j in 1:nsite){
```

```
 sp.rich[j] <- sum(Z[j,]) # Number of species
}


}
```

Table S3. Formulation of Bayesian hierarchical generalized linear mixed models for apple quality characteristics as a function of landscape enhancements and intensity of pesticide use implemented in JAGS for the apple data in 2017-2019 in southern Québec, Canada.

```
##linear mixed model for diameter, mass, and sugar level

##Apple condition for Gaussian response

model {
for (i in 1:nsites){
   alpha.site[i] ~ dnorm(mu.site, tau.site)
}

##hyperparameters for random intercepts
mu.site ~ dnorm(0, 0.001)
sigma.site ~ dunif(0, 100)
tau.site <- 1 / (sigma.site * sigma.site)

##fixed effects
beta.managed ~ dnorm(0, 0.001)   #managed = 0, 1
beta.intensity ~ dnorm(0, 0.001) #intensity <= 48.5 (0), > 48.5 (1)

##residual variance
sigma ~ dunif(0, 100)
tau <- 1/(sigma * sigma)

##likelihood
for (i in 1:n) {
   Mass[i] ~ dnorm(mu[i], tau)
   mu[i] <- alpha.site[Site.num[i]] + beta.managed * Managed[i] + beta.intensity *
Intensity[i]
}



##generalized linear mixed model for total number of seeds
##Apple condition for Poisson response
model {
for (i in 1:nsites){
   alpha.site[i] ~ dnorm(mu.site, tau.site)
```

```
}

##hyperparameters for random intercepts
mu.site ~ dnorm(0, 0.001)
sigma.site ~ dunif(0, 100)
tau.site <- 1 / (sigma.site * sigma.site)

##fixed effects
beta.managed ~ dnorm(0, 0.001)   #managed = 0, 1
beta.intensity ~ dnorm(0, 0.001) #intensity <= 48.5 (0), > 48.5 (1)

##likelihood
for (i in 1:n) {
   Seed[i] ~ dpois(lambda[i])
   log(lambda[i]) <- alpha.site[Site.num[i]] + beta.managed * Managed[i] +
beta.intensity * Intensity[i]
}

}
```

References

1. SAgE Pesticides Traitements phytosanitaires et risques associés Available online: https://www.sagepesticides.qc.ca/Recherche/RechercheTraitement.
